# Reclaiming the Smile: Full Mouth Rehabilitation of a Generalized Attrition Patient Using the Hobo Twin-Stage Technique

**DOI:** 10.7759/cureus.39260

**Published:** 2023-05-20

**Authors:** Ashish Dadarwal, Vineet Sharma, Kriti Sareen, Dharmendra K Vashistha, Rahul Madaan

**Affiliations:** 1 Prosthodontics, Rajasthan University of Health Sciences (RUHS) College of Dental Sciences, Jaipur, IND; 2 Oral Medicine and Radiology, Rayat Bahra Dental College and Hospital, Mohali, IND; 3 Oral and Maxillofacial Pathology, Rajasthan University of Health Sciences (RUHS) College of Dental Sciences, Jaipur, IND

**Keywords:** full mouth rehabilitation, vertical dimension of occlusion, generalized attrition, porcelain fused to metal crowns, hobo twin-stage procedure

## Abstract

This clinical report details the successful rehabilitation of a 63-year-old male patient with severe tooth wear, a reduced vertical dimension of occlusion, and esthetic concerns. The Hobo twin-stage procedure addressed these issues while improving the patient's oral health and quality of life. After ensuring adequate oral hygiene, the treatment began with scaling and root planning, followed by diagnostic impressions. An occlusal splint was fabricated, followed by a diagnostic wax-up and tooth preparation. Full-arch impressions of prepared teeth were made using the addition of silicon elastomeric impression material, and chairside provisional crowns were fabricated. The working casts were mounted on a semi-adjustable articulator, and the metal copings were tried on before being built up in porcelain. The patient achieved successful outcomes and expressed satisfaction with the treatment. The Hobo twin-stage technique and porcelain-fused-to-metal crowns can be viable approaches for restoring the teeth's form and function while enhancing the patient's oral health and esthetics. However, regular follow-up appointments and good oral hygiene maintenance are essential for the long-term success of the treatment.

## Introduction

The loss of natural tooth structure due to generalized attrition is a significant challenge in restorative dentistry [1]. Although gradual wear of the occlusal surfaces of teeth is a natural occurrence over a person's lifetime, excessive wear can result in several problems, such as pulpal injury, occlusal disharmony, compromised function, and esthetic deformities. In such cases, a comprehensive treatment plan is required to address the functional and occlusal aspects and esthetic concerns [2]. Restoring the lost tooth structure using porcelain-fused-to-metal (PFM) crowns can be a viable option to restore the form and function of the teeth while improving overall oral health and esthetics.

This clinical report highlights the use of the Hobo twin-stage procedure to rehabilitate a patient suffering from generalized attrition and reduced vertical dimension of occlusion (VDO). The Hobo twin-stage technique creates anterior guidance to achieve a pre-planned and harmonious posterior disocclusion in line with the condylar path [3,4]. The treatment aimed to restore esthetics, function, and occlusion while improving the patient's oral health and quality of life. The successful outcome of this treatment not only improved the patient's appearance and function but also instilled confidence and satisfaction [4].

## Case presentation

**Figure 1:**
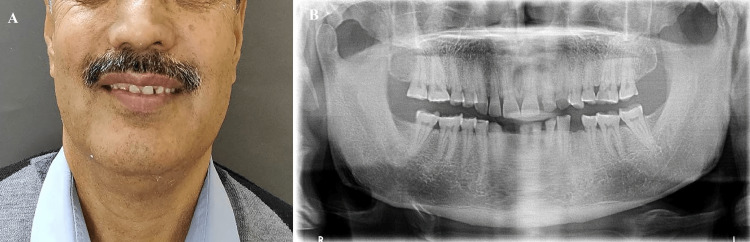
(A) Preoperative extraoral portrait view; (B) panoramic radiograph

**Figure 2:**
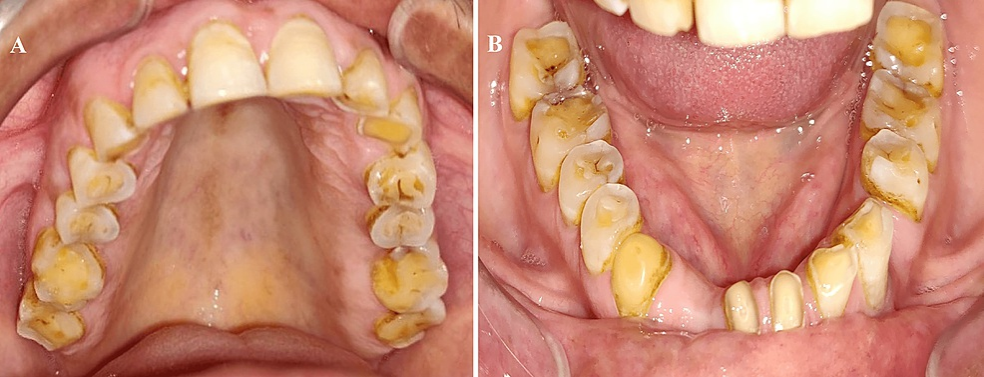
Preoperative photograph showing generalized attrition: (A) maxillary arch; (B) mandibular arch

A 63-year-old male presented to the Department of Prosthodontics with a chief complaint of worn-out teeth and difficulty chewing. He had no significant medical history and reported no known allergies or adverse drug reactions. He was cooperative and willing to undergo treatment. Clinical examination revealed severe generalized attrition of all teeth and a decreased VDO (Figures 1-2). The patient had a decayed maxillary left first molar (26), which was subsequently restored using amalgam. The mandibular left first premolar (34) and right lateral incisor (42) were missing, and the mandibular left central incisor (31) was extracted later. The oral hygiene was poor, with calculus deposits and gingivitis. The patient's temporomandibular joint was examined and was functioning normally with no pain or discomfort. The amount of freeway space was determined to be 5 millimeters (mm), and it was determined that the VDO could be restored by raising it by 2 mm. The patient was informed about the diagnosis, and a detailed discussion was held to explain the treatment plan. The patient was advised to undergo scaling and root planing to improve oral hygiene, followed by a gingivectomy of maxillary anterior teeth, to produce a gingival zenith before initiating the restorative treatment. The patient provided informed consent, and the treatment was initiated accordingly.

The treatment plan for the patient with severe tooth wear and reduced VDO included full mouth restorations using either the Hobo twin-stage or Pankey-Mann Schuyler technique. Ultimately, the decision was made to use the Hobo twin-stage procedure for full mouth rehabilitation with PFM crowns. This was chosen due to the advantages of single-step tooth preparation, pre-set values, and the absence of the need for condylar and lateral records.

Clinical procedure

**Figure 3:**
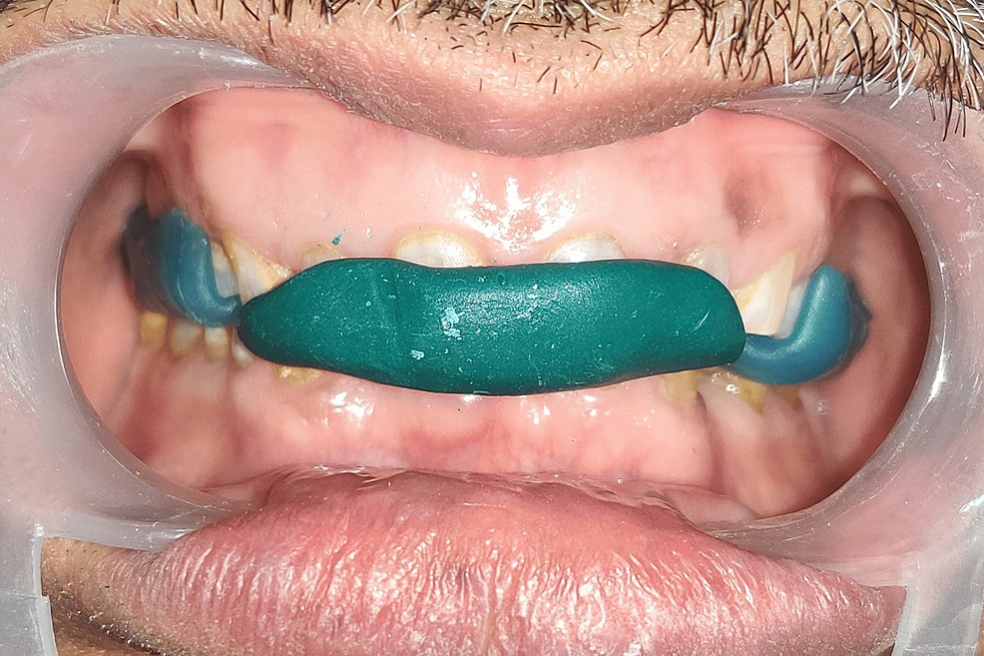
Centric record at raised vertical dimension

The procedure began with scaling and root planing to promote dental hygiene and periodontal health. Gingivectomy was performed to establish the gingival zenith distal to the long axis of the maxillary central and lateral incisors and along the long axis of the maxillary canines. Diagnostic impressions were made after ensuring optimal dental hygiene and complete healing of the tissues. A facebow record was made and transferred to the semi-adjustable articulator (Hanau™ Wide-Vue; Whip Mix Corporation, Louisville, USA). The next step involved making a centric record and using it to mount the mandibular cast onto the articulator (Figure 3).

**Figure 4:**
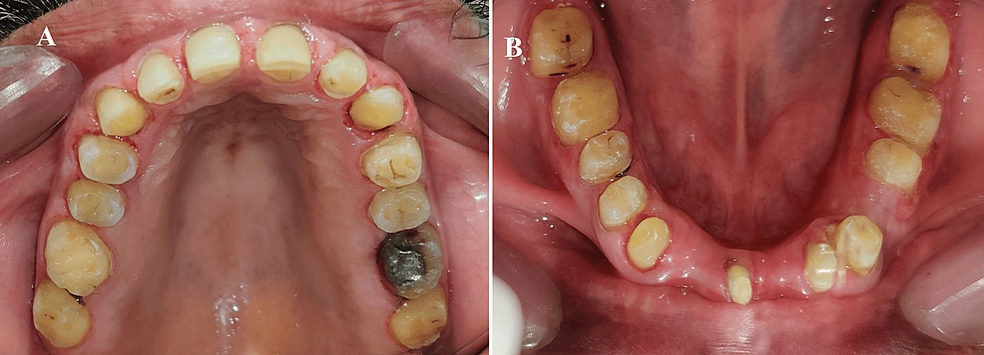
Tooth preparation: (A) maxillary arch; (B) mandibular arch

**Figure 5:**
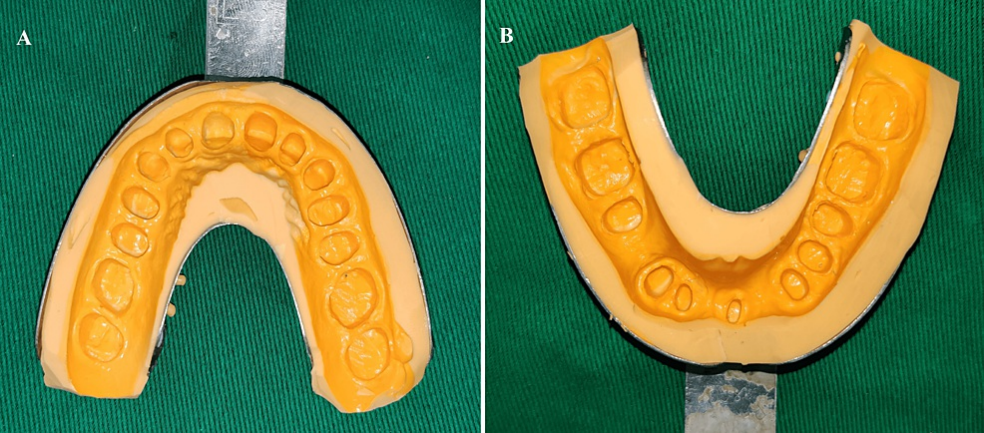
Master impression with poly siloxane impression material: (A) maxillary arch; (B) mandibular arch

Afterward, the patient's adaptability to the new VDO was observed for six weeks using an occlusal splint at a 2 mm increase in VDO. A diagnostic wax-up and a putty index followed this. The teeth were then prepared according to the standard protocol for PFM restoration with minimal occlusal reduction (0.3-0.5 mm), and a complete arch impression was made with elastomeric impression material (GC Flexceed; GC India, Telangana, India) (Figures 4-5). Using the putty index from the diagnostic wax-up, provisional crowns were fabricated.

**Figure 6:**
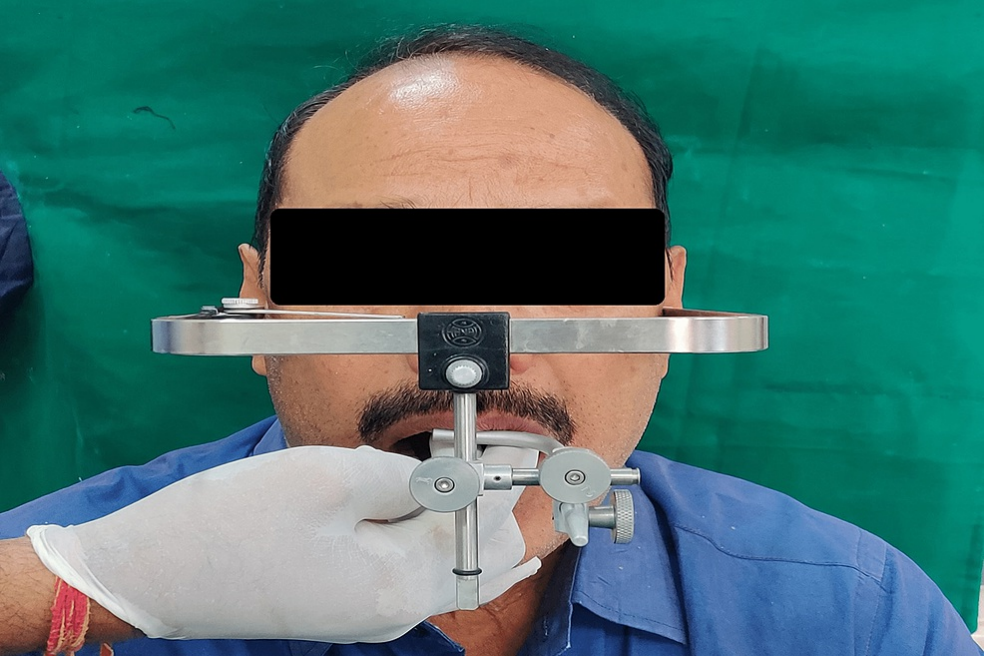
Facebow record

A facebow record was made and the working casts were mounted on a semi-adjustable articulator with the facebow (Figure 6).

**Figure 7:**
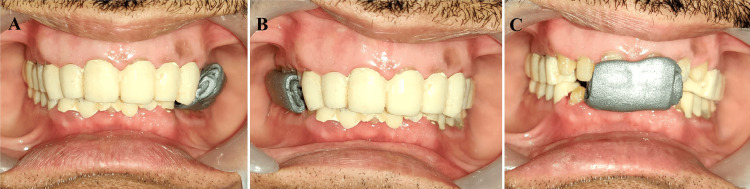
Segmental inter-occlusal records to transfer VDO and CR: (A) left posterior; (B) right posterior; (C) anterior

To transfer the VDO and CR, the temporary provisional crowns from the left posterior section were removed. In contrast, the temporary provisional crowns from the right and anterior sections were left intact. A piece of record material (Alu Wax; I-Med Dental Solutions, Vadodara, India) was placed between the prepared teeth on the left posterior section. The same procedure was repeated in the left and anterior sections, and the mandibular cast was mounted using three segmental interocclusal records (Figure 7).

**Figure 8:**
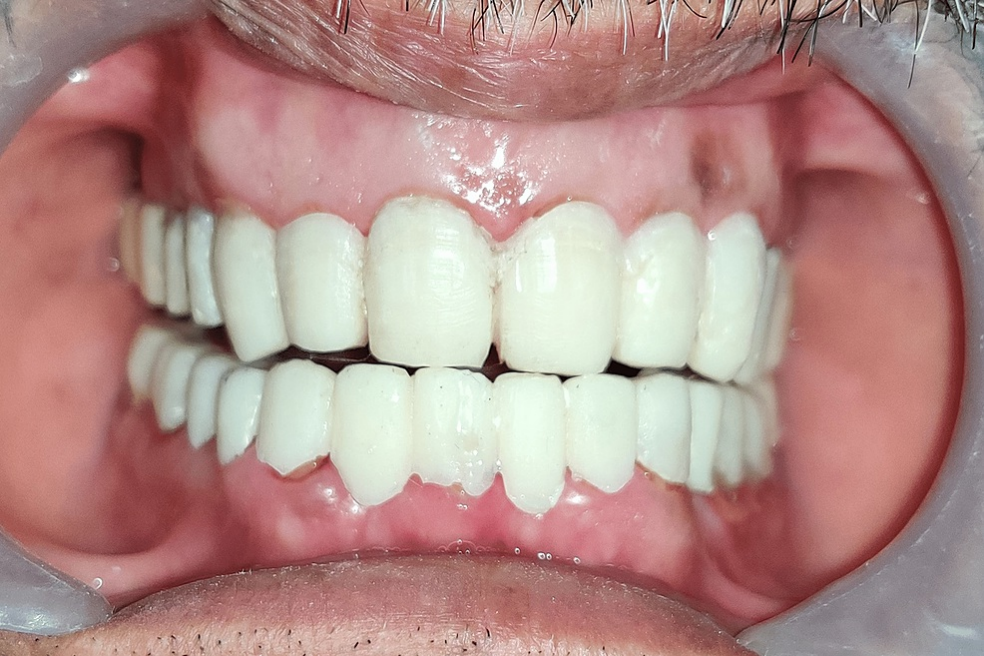
Provisional crowns in situ

Finally, non-eugenol temporary cement (NETC; Meta Biomed, Colmar, USA) was used to cement the temporary provisional crowns (Figure 8).

**Figure 9:**
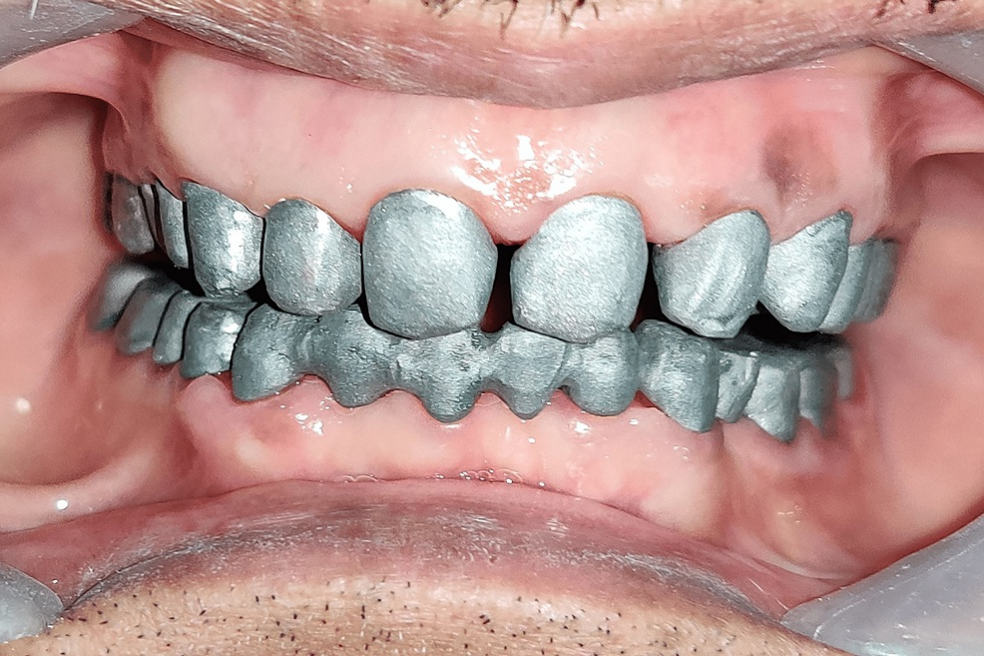
Metal copings try in

After creating wax patterns and casting them, metal copings were tried in the patient's mouth to ensure a proper fit (Figure 9). Subsequently, the copings were fitted onto the working cast that was attached to the articulator, and porcelain build-up was carried out in two phases. The first phase involved removing the anterior section of the cast and adjusting the condylar and incisal guidance of the articulator to conform with Hobo's condition 1, which ensures standard effective cusp angles. The occlusal morphology of the posterior teeth was built up so that the maxillary and mandibular cusps came into contact during eccentric movement, resulting in a balanced articulation and standard cusp angle for each cusp.

In the second phase, the anterior section was reattached to the cast, and the condylar and incisal guidance was set to Hobo's condition 2 to achieve posterior disocclusion (Table 1).

**Table 1:** Articular adjustment values in the Hobo twin-stage technique

Condition	Condylar path		Anterior guide table	
	Sagittal condylar path inclination	Bennet angle	Sagittal inclination	Lateral wing angle
Condition 1: without anterior teeth	25 degrees	15 degrees	25 degrees	10 degrees
Condition 2: with anterior teeth	40 degrees	15 degrees	45 degrees	20 degrees

**Figure 10:**
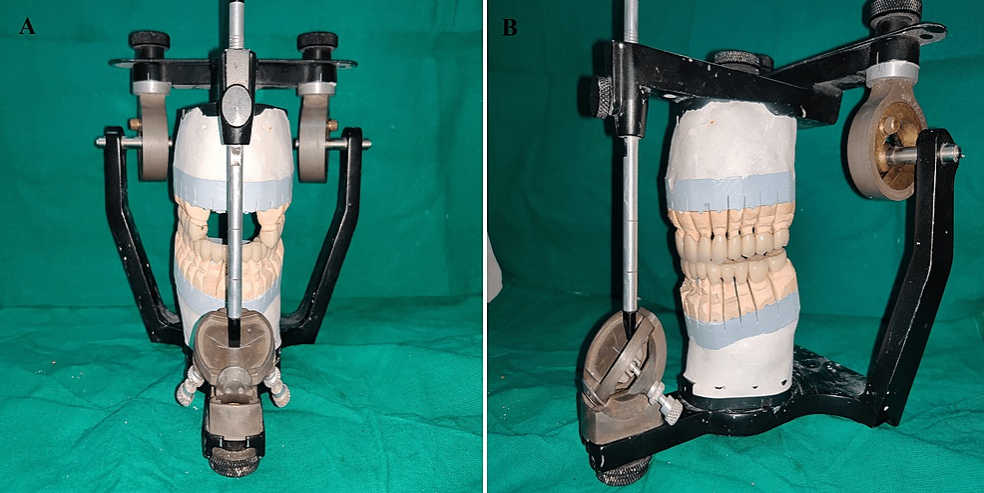
(A) Condition 1 without anterior segment; (B) condition 2 with anterior segment showing posterior disocclusion

Porcelain build-up for the anterior teeth was then carried out to ensure that the maxillary and mandibular incisors were contacted during protrusive movement and that the maxillary and mandibular canines on the working side came into contact during lateral movement. As a result, the anterior guidance was established, and the standard amount of disocclusion was produced (Figure 10).

**Figure 11:**
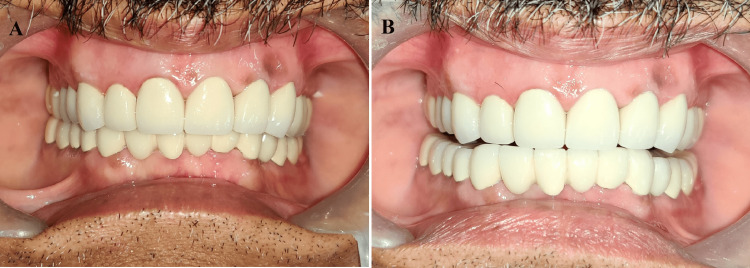
Final prostheses in situ: (A) centric occlusion; (B) posterior disocclusion in protrusion

**Figure 12:**
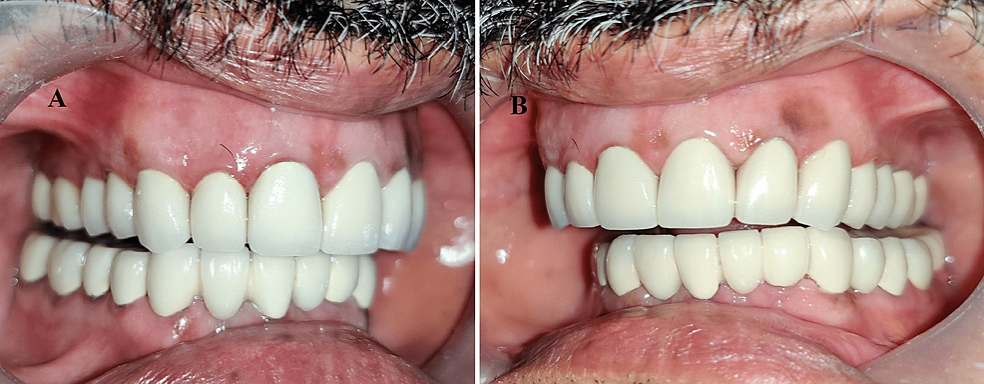
Posterior disocclusion in the eccentric movement: (A) right eccentric; (B) left eccentric

**Figure 13:**
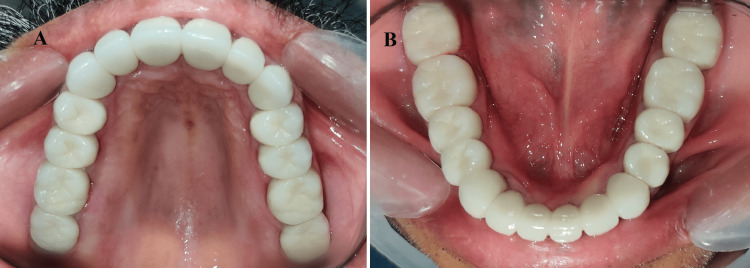
Occlusal view of final PFM prostheses: (A) maxillary arch; (B) mandibular arch PFM: porcelain fused to metal

**Figure 14:**
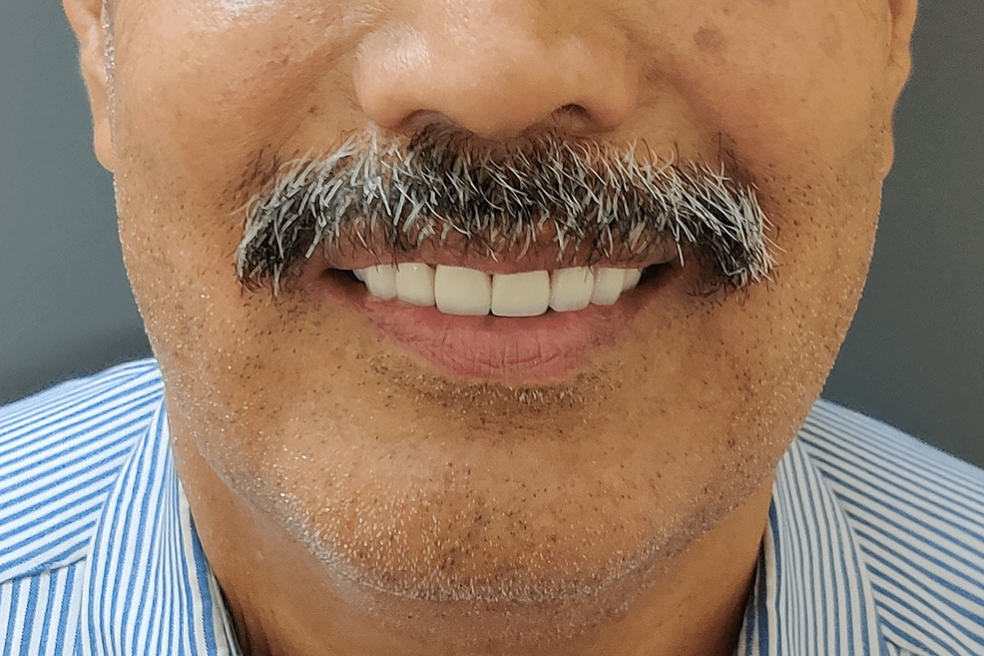
Smile of satisfaction

Finally, the PFM crowns were cemented in place using polycarboxylate cement (Figure 11-14). The occlusion was adjusted to achieve proper contact and function. The patient was instructed on maintaining good oral hygiene and scheduled for regular follow-up appointments to monitor the healing process and ensure the long-term success of the treatment. The patient was pleased with the final result and reported a significant improvement in the esthetics and function of the teeth. The patient's confidence and self-esteem were also restored. The patient was advised to maintain good oral hygiene and attend regular follow-up appointments to ensure the long-term success of the treatment.

## Discussion

Generalized attrition is a common problem among older adults, leading to functional, esthetic, and occlusal concerns [3]. The restoration of lost tooth structure using PFM crowns is a viable option to restore the form and function of teeth while improving oral health and esthetics [5]. In this clinical report, we discussed using the Hobo twin-stage procedure to rehabilitate a patient with severe generalized attrition, resulting in a reduced VDO. The Hobo twin-stage technique developed by Hobo and Takayama is a comprehensive approach that establishes anterior guidance to create a predetermined and harmonious posterior disocclusion with the condylar path [6-8]. Hobo's approach is distinguished by its ability to deliver a reliable posterior disclusion and anterior guidance that adheres to the condylar path. This method allows for the precise creation of cuspal angle and anterior guidance without considering the remaining natural teeth, as all teeth are restored in full mouth rehabilitation. Additionally, the programmed disocclusion amount can be accurately reproduced [6,7].

The fundamental principles of the Hobo twin-stage technique require the establishment of adjustment values for an articulator to replicate a standard cusp angle and a standard amount of disocclusion. To achieve conditions 1 and 2, specific calculations are used to select the necessary adjustment values for the articulator. The standard value for the sagittal protrusive effective cusp angle is 25 degrees; thus, it is recommended to adjust both the condylar path and anterior guide table to 25 degrees and fabricate cusps without anterior teeth to achieve a balanced articulation. This enables uniform fabrication of the 25-degree cusp angle, which is the adjustment value required for the articulator to attain condition 1. Once the cusps have been waxed to the standard angle value, the anterior guidance should be established to replicate a standard amount of disocclusion. To generate the standard amount of disocclusion on molars and fabricate physiological anterior guidance, a combination of 40 degrees for the condylar path and 45 degrees for the anterior guide table is used. This is the adjustment value for the articulator required to achieve condition 2 [6,7].

In 1984, Turner and Missirlian proposed a classification system for the treatment of severely worn dentition based on the degree of loss of VDO and the amount of available space for restoration [9]. This classification system has been widely utilized with conventional treatment approaches involving multiple crown-lengthening procedures to increase VDO. In addition to these procedures, occlusal splints have been used as a reversible and conservative diagnostic tool to assess the patient's adaptation to the altered VDO. In the present case, the patient was closely monitored for six weeks to determine adaptation to the removable occlusal splint, and adaptation to the provisional restoration was observed for two months [10,11].

Using provisional restorations allowed for evaluating the restored VDO before the final restorations were fabricated. This ensured that any discrepancies or discomfort in the new occlusal relationship could be addressed before the final restorations were cemented. The use of provisional restorations also allowed for the development of proper occlusal contacts and tooth contours, which were transferred to the final restorations [12,13].

Various occlusal schemes have been proposed for full mouth rehabilitation, such as PMS, Hobo's twin table, the Hobo twin-stage technique, Youdelis, and Nyman and Lindhe Scheme [3,14]. Among these approaches, PMS and the Hobo twin-stage technique are the most popular. The Hobo twin-stage technique is simpler and easier to understand and is highly predictable in terms of achieving functional and esthetic outcomes. The Hobo twin-stage technique can be completed in a shorter amount of time and is more flexible than the PMS technique. The Hobo twin-stage technique can be modified and adapted to fit the specific needs and goals of each patient, while the PMS technique is more rigid and prescriptive. The Hobo twin-stage technique can be less expensive than the PMS technique because it requires fewer appointments and less time in the dental chair [8,15]. The Hobo twin-stage technique has preset values and no need for condylar and lateral records. Overall, the Hobo twin-stage technique is a more straightforward, predictable, efficient, flexible, and cost-effective dental treatment planning method.

PFM crowns, in this case, allowed for the restoration of the lost tooth structure while improving the esthetic appearance of the teeth [5]. Using porcelain also ensures the longevity of the rehabilitation and provides excellent marginal adaptation and surface finish. The patient reported high satisfaction with the esthetic outcome of the treatment, as well as the improvement in the function and occlusion of the teeth. However, it is important to note that regular follow-up appointments and good oral hygiene maintenance are essential for the long-term success of the treatment.

## Conclusions

This case report illustrated the successful application of the Hobo twin-stage technique for full-mouth rehabilitation using PFM crowns to address a patient's severe tooth wear and reduced VDO. The treatment provided a viable option for restoring the form and function of the teeth while improving the patient's oral health and esthetics. The careful planning, accurate preparation, and meticulous execution of the treatment resulted in a successful outcome, instilling confidence and satisfaction in the patient. The Hobo twin-stage procedure can significantly improve the outcomes of restorative dentistry and provide long-lasting benefits to patients with complex dental problems. However, each patient's case is unique, and careful planning and a comprehensive approach should be taken to determine the optimal treatment plan.

## References

[1] Warreth A, Abuhijleh E, Almaghribi MA, Mahwal G, Ashawish A (2020). Tooth surface loss: a review of literature. Saudi Dent J.

[2] Thirumurthy VR, Bindhoo YA, Jacob SJ, Kurien A, Limson KS, Vidhiyasagar P (2013). Diagnosis and management of occlusal wear: a case report. J Indian Prosthodont Soc.

[3] Thimmappa M, Katarya V, Parekh I (2021). Philosophies of full mouth rehabilitation: A systematic review of clinical studies. J Indian Prosthodont Soc.

[4] Maharjan A, Joshi S, Verma A, Rimal U (2019). Rehabilitation of severely attrited teeth with Hobo twin stage technique: a case report. JNMA J Nepal Med Assoc.

[5] Christensen GJ (1986). The use of porcelain-fused-to-metal restorations in current dental practice: a survey. J Prosthet Dent.

[6] Hobo S (1991). Twin-tables technique for occlusal rehabilitation: part I--mechanism of anterior guidance. J Prosthet Dent.

[7] Hobo S (1991). Twin-tables technique for occlusal rehabilitation: part II--clinical procedures. J Prosthet Dent.

[8] Tiwari B, Ladha K, Lalit A, Dwarakananda Naik B (2014). Occlusal concepts in full mouth rehabilitation: an overview. J Indian Prosthodont Soc.

[9] Turner KA, Missirlian DM (1984). Restoration of the extremely worn dentition. J Prosthet Dent.

[10] Ganddini MR, Al-Mardini M, Graser GN, Almog D (2004). Maxillary and mandibular overlay removable partial dentures for the restoration of worn teeth. J Prosthet Dent.

[11] Jain AR, Nallaswamy D, Ariga P, Philip JM (2013). Full mouth rehabilitation of a patient with reduced vertical dimension using multiple metal ce ramic restorations. Contemp Clin Dent.

[12] LeSage BP (2020). CAD/CAM: applications for transitional bonding to restore occlusal vertical dimension. J Esthet Restor Dent.

[13] Song MY, Park JM, Park EJ (2010). Full mouth rehabilitation of the patient with severely worn dentition: a case report. J Adv Prosthodont.

[14] Ram S, Shah N, Nadgere J, Iyer J, Ezzy H, Gaikwad A (2019). Full-mouth rehabilitation of worn dentition by hobo twin-stage philosophy: a case series. J Contemp Dent.

[15] Sharma V, Paliwal J, Meena KK, Dadarwal A, Singla A (2021). Full mouth rehabilitation of a patient with amelogenesis imperfecta using twin stage procedure. J Clin Diagn Res.

